# sTREM-1 as a biomarker for sepsis diagnosis and prognosis following abdominal surgery

**DOI:** 10.1093/labmed/lmaf074

**Published:** 2025-11-22

**Authors:** Patricia Bautista-Carbajal, Juan Pedro Chávez-Pérez, Miguel Leonardo García-León, José Arturo Cabrera-Sánchez, Neyla Baltazar-López, Felipe Rafael Zaldivar-Ramirez, Rosa María Wong-Chew

**Affiliations:** Facultad de Medicina, Infectious Diseases Research Laboratory, Research Division, Universidad Nacional Autónoma de México, Ciudad de México, México; Surgery Department, Intensive Care Unit and Central Laboratory, Hospital General de México “Dr Eduardo Liceaga,” Ciudad de México, México; Facultad de Medicina, Infectious Diseases Research Laboratory, Research Division, Universidad Nacional Autónoma de México, Ciudad de México, México; Facultad de Medicina, Infectious Diseases Research Laboratory, Research Division, Universidad Nacional Autónoma de México, Ciudad de México, México; Surgery Department, Intensive Care Unit and Central Laboratory, Hospital General de México “Dr Eduardo Liceaga,” Ciudad de México, México; Surgery Department, Intensive Care Unit and Central Laboratory, Hospital General de México “Dr Eduardo Liceaga,” Ciudad de México, México; Facultad de Medicina, Infectious Diseases Research Laboratory, Research Division, Universidad Nacional Autónoma de México, Ciudad de México, México

**Keywords:** after surgery, sepsis, sTREM1, diagnosis and prognosis

## Abstract

**Introduction:**

Sepsis represents a critical response to infection; it is characterized by systemic inflammation, shock, and potential organ failure. Soluble triggering receptor expressed on myeloid cells 1 (sTREM-1) has been identified as a crucial marker in sepsis, connecting the activation of innate immunity to systemic inflammation.

**Methods:**

This prospective nested case control study was carried out in the intensive care unit and surgery department from March 2018 to June 2019. Adults undergoing abdominal surgery, with and without sepsis, were included in the study, and sTREM-1 and cytokine levels were measured.

**Results:**

A total of 120 patients were included in the study, comprising 31 noninfected individuals, 37 with sepsis, and 52 with septic shock. sTREM-1 levels were statistically significantly elevated in patients with sepsis and septic shock compared with noninfected individuals (*P* < .001). Receiver operating characteristic curve analysis revealed an area under the curve of 0.722 for sTREM-1 in the prediction of septic shock.

**Discussion:**

Elevated sTREM-1 levels are associated with the severity of sepsis and may function as a prognostic biomarker. Additional research is required to confirm these findings and investigate therapeutic interventions aimed at the sTREM-1 pathway.

## Introduction

Sepsis represents a critical response to infection; it is characterized by systemic inflammation, which can lead to shock and organ failure. The intricate interplay between infection and the host immune response plays a pivotal role in the pathogenesis of sepsis, necessitating the identification of biomarkers for early diagnosis and deeper understanding of underlying immune mechanisms. Soluble triggering receptor expressed on myeloid cells 1 (sTREM-1) has emerged as a biomarker, amplifying the immune response in sepsis and linking innate immunity activation to the systemic inflammation characteristic of this condition.

TREM-1 and its soluble variant sTREM-1 rise in sepsis and chronic inflammatory states and appear to amplify inflammatory signals.^1^ In studies of *Streptococcus pyogenes* sepsis, sTREM-1 levels ranged from 257 to 3075 pg/mL and correlated with bacterial load and disease severity.^2^ Elevated sTREM-1 and high sTREM-1/tumor necrosis factor α (TNF-α) ratios corresponded with poor outcomes in ventilator-associated pneumonia and septic shock.^3^ Activation of membrane-bound TREM-1 promoted proinflammatory signaling, inducing cytokines such as interleukin 6 (IL-6), TNF-α, and IL-8, as demonstrated in models of sepsis and inflammatory bowel disease.^4^

Interventions that modulate TREM-1 signaling offer a nuanced effect.^2^ Use of decoy receptors, antagonistic peptides (eg, LP17, M3), or partial gene silencing reduced cytokine release, tissue injury, and (in some experiments) improved survival (for instance, survival increased from 45% to 80% in 1 sepsis model).^2^ In contrast, complete silencing impaired bacterial clearance and increased mortality, emphasizing that effective modulation requires a calibrated approach.^5^ These findings indicate that the interplay between sTREM-1 and TREM-1 intensifies inflammatory responses in acute sepsis and chronic inflammation and that targeted modulation can alter these responses within a narrow therapeutic window.^2^

Research indicates that sTREM-1 levels are substantially elevated in patients with sepsis, from neonates to adults, correlating with disease severity and mortality risk. The role of sTREM-1 in modulating the inflammatory response suggests its potential as a biomarker for distinguishing septic from nonseptic inflammation.^6^^,^^7^

Moreover, integrating sTREM-1 with other biomarkers, such as the soluble form of ST2 and procalcitonin, has enhanced the accuracy of sepsis diagnosis and prognosis.^8^ This multibiomarker approach contributes to a more comprehensive understanding of patient conditions, facilitating personalized treatment strategies. The prognostic value of sTREM-1 in pediatric sepsis and its role in risk stratification among febrile children underscore its importance in immune surveillance and infection response.^9^^,^^10^ These findings highlight the potential of sTREM-1 in mitigating sepsis-induced immune dysregulation and improving risk assessment and clinical management strategies.

This study focused on the diagnostic and prognostic role of sTREM-1 within a defined postoperative cohort of surgical patients. Additional inflammatory mediators were explored in a supplementary analysis to provide contextual understanding of the immune environment. By synthesizing existing research on the diagnostic and prognostic relevance of sTREM-1 across various immunologic contexts, this study sought to advance the understanding of sepsis pathophysiology and improve patient outcomes.^11–15^

## Methods

### Study design and population

This research was a prospective, nested case control study within a cohort translational study, conducted in the intensive care unit (ICU) and the Department of Surgery at Hospital General de Mexico “Dr Eduardo Liceaga” from March 2018 to June 2019. The study was approved by the Ethics and Research committees of the Hospital General de Mexico (DI/18/202/03/003) and the Faculty of Medicine at Universidad Nacional Autónoma de México (FM/DI/SR/037/2018). Patients were directly approached for participation, or their guardians were contacted when consent could not be obtained. Written informed consent was obtained from participants or their legal representatives. This study adhered to Good Clinical Practice guidelines and standards and the International Conference for Harmonisation of Technical Requirements for Pharmaceuticals for Human Use.

### Inclusion and exclusion criteria

The inclusion criteria encompassed adult patients, including individuals undergoing uncomplicated abdominal surgery and patients diagnosed with abdominal sepsis or septic shock according to internationally accepted clinical definitions consistent with the Third International Consensus Definitions for Sepsis and Septic Shock consensus. Specifically, sepsis was characterized by acute organ dysfunction associated with a dysregulated host response to infection, while septic shock was identified by persistent hypotension requiring vasopressors to maintain a mean arterial pressure of 65 mm Hg or higher and serum lactate levels above 2 mmol/L, despite adequate fluid resuscitation.^16^ Exclusion criteria included individuals younger than 18 of age or older than 65 years of age; individuals with concurrent infectious diseases, disseminated cancer, pregnancy, or AIDS; persons on immunosuppressive therapy; or lack of consent.

### Data collection and biological sampling

Clinical data were recorded, including demographics, primary diagnosis, admission type, mortality rates, duration of mechanical ventilation and intensive care, discharge outcomes, Brussels and sequential organ failure assessment (SOFA) scores, routine blood tests, and body temperature. Blood specimens were collected in yellow cap tubes preoperatively and on day 2 from patients without complications as well as at hospital admission and on days 2 and 5 for patients with sepsis or septic shock. Following collection, blood samples were centrifuged, and serum was stored at −70 °C for subsequent analysis. EDTA tubes were obtained for immediate cellular analysis.

### Calculation of clinical scores

The SOFA score was calculated using standard criteria across 6 organ systems (respiratory, cardiovascular, hepatic, coagulation, renal, and neurologic), assigning scores from 0 to 4 for each based on routine laboratory values and clinical parameters recorded at each time point. The Brussels score was derived according to the institutional protocol in use at the Hospital General de México at the time of the study (2018-2019), based on a point system evaluating organ dysfunction in ICU patients. Although this scoring system is no longer in widespread use, it was retained in this analysis to maintain consistency with historical datasets and internal benchmarking practices.

### sTREM-1 and cellular TREM-1 assays

Serum levels of sTREM-1 were quantified in duplicate using the Human TREM-1 Quantikine ELISA Kit (R&D Systems, catalog No. DTRM10B), following the manufacturer’s instructions. The assay has an analytical measuring range of 62.5 to 4000 pg/mL. Sensitivity, expressed as the minimum detectable dose, ranged from 3.88 to 30.6 pg/mL across 40 assays, with a mean minimum detectable dose of 13.8 pg/mL. Intra-assay and interassay coefficients of variation (s/x̄) × 100 were 3.6% to 5.2% and 5.8% to 7.4%, respectively. No manufacturer-provided reference ranges were available for healthy individuals; therefore, the mean (SD) value in the noninfected group (102 [20] pg/mL) was used as an internal reference for comparison. Peripheral blood mononuclear cells (PBMCs) were isolated using Fycoll-paque. TREM-1 expression on monocytes and neutrophils was assessed in a subgroup of 19 surgical patients: 10 without complications, 4 with sepsis, and 5 with septic shock. Flow cytometry was performed using a panel of fluorescently tagged antibodies targeting specific lineage markers. CD64 and CD16 were used as monocyte/macrophage markers, while CD66b and CD16 were used to identify neutrophils. Anti-TREM-1 (CD354-PE) staining was included to evaluate receptor expression. The PBMCs were stained in the dark for 30 minutes at 4 °C, washed with phosphate-buffered saline, and resuspended in fluorescence activated cell sorting buffer before acquisition. Instrument calibration and compensation controls were performed daily using unstained cells, single-stained controls, and compensation beads to ensure appropriate gating and fluorescence separation. Data acquisition was carried out on an Attune NxT Flow Cytometer (Thermo Fisher Scientific), and FlowJo, version 10, software (TreeStar/Becton Dickinson) was used for data processing at the Infectious Diseases Research Laboratory of the Faculty of Medicine, National Autonomous University of Mexico.

### Cytokine detection

T helper 1 (Th1)/Th2 cytokines were quantified by flow cytometry using the LEGENDplex Human Th1/Th2 Panel (BioLegend, catalog No. 741030). The panel includes detection of IL-2, IL-4, IL-5, IL-6, IL-10, IL-13, interferon γ (IFN-γ), and TNF-α. The assay was performed according to the manufacturer’s protocol. The analytical measuring range extended up to 10 000 pg/mL. The lower limits of detection (LODs) in serum or plasma matrix for each analyte were as follows: IL-2 (1.20 pg/mL), IL-4 (2.30 pg/mL), IL-5 (2.40 pg/mL), IL-6 (2.20 pg/mL), IL-10 (1.70 pg/mL), IL-13 (5.30 pg/mL), IFN-γ (3.40 pg/mL), and TNF-α (3.70 pg/mL), as reported by the manufacturer. Values below these thresholds were reported as “<LOD” for the respective cytokine. The assay’s performance was validated through use of internal controls, standard curves, and duplicate analysis of all samples.

### Statistical analysis

Univariate analysis was employed to conduct descriptive statistics. Comparisons of TREM-1 expression on neutrophils and monocytes as well as serum sTREM-1 levels were conducted using analysis of variance. Spearman rank correlation was used to assess correlations between membrane-bound and soluble TREM-1, while Pearson correlation was applied to examine relationships between SOFA and Brussels scores and serum sTREM-1 levels. Receiver operating characteristic (ROC) curves were generated to evaluate the predictive value of sTREM-1 for septic shock, with statistical significance set at *P* < .05. All analyses were performed using SPSS, version 22, software (IBM Corp).

## Results

### Demographic and clinical characteristics

This research included the demographic and clinical characteristics of 120 patients who had undergone abdominal surgery, categorized into 3 distinct postoperative condition groups: noninfected (31 patients), sepsis (37 patients), and septic shock (52 patients). The mean (SD) age was 45.87 (12) years for noninfected patients, 40.11 (12) years for patients with sepsis, and 48.23 (14) years for patients with septic shock (*P* = .07).

Sex distribution exhibited statistically significant differences (*P* = .001), with a markedly higher proportion of male patients in the sepsis (55%) and septic shock (44%) groups compared with the noninfected group (10%).

Statistically significant differences were observed in physical characteristics—specifically, weight (*P* = .04) and height (*P* = .001), indicating that patients with sepsis and septic shock tended to be heavier and taller. Body mass index, however, did not show statistically significant differences among the groups (*P* = .68).

The prevalence of comorbidities was statistically significantly higher in the septic shock group (57%) than in the sepsis (29%) and noninfected (35%) groups (*P* = .047). Mortality rates were notably higher in the septic shock group at 32% compared with 0% in the other groups (*P* = .004), underscoring the severe impact of septic shock on patient survival.

Laboratory findings at the time of hospital admission provided further insights into patients’ conditions. Mean (SD) white blood cell counts were statistically significantly higher in the sepsis (14.97 [5.55] 10^3^/µL) and septic shock (16.83 [9.56] 10^3^/µL) groups than in the noninfected group (7.67 [1.75] 10^3^/µl) (*P* < .001), indicating an inflammatory response to infection. Mean (SD) hemoglobin levels were statistically significantly lower in the septic shock group (10.23 [1.95] g/dL), correlating with disease severity (*P* < .001). Platelet counts did not show a statistically significantly difference among groups (*P* = .94).

The distribution of surgical procedures varied significantly among the groups (*P* < .001), reflecting the clinical context of each patient’s condition. Laparoscopic cholecystectomy was predominantly performed in noninfected patients (81%), while appendectomies (37% and 31%) and intestinal resections (30% and 26%) were more common in sepsis and septic shock patients, respectively (Table 1).

**Table 1.** Demographic and Clinical Characteristics of Patients Who Underwent Abdominal Surgery and Were Noninfected, Had Sepsis, and Had Septic Shock

Variable	Noninfected (*n* = 31)	Sepsis (*n* = 37)	Septic shock (*n* = 52)	***P* value**
Age, mean (SD), y	45.87 (12.76 )	40.11 (12.48)	48.23 (14.92)	.070
Female sex, No. (%)	28 (90)	17 (46)	29 (56)	.001
Male sex, No. (%)	3 (10)	20 (55)	23 (44)	
Weight, mean (SD), kg	67.40 (9.28 )	77.91 (19.96)	72.50 (16.49)	.037
Height, mean (SD), m	1.56 (0.07)	1.64 (0.09)	1.61 (0.1)	.001
Body mass index, mean (SD)	27.78 (4.46)	28.75 (5.78)	28.07 (6.49)	.680
Comorbidities, No. (%)	11 (35)	11 (29)	29 (57)	.047
Deaths, No. (%)	0 (0)	0 (0)	17 (32)	.004
White blood cell count at admission, mean (SD), ×10^3^/µL	7.67 (1.75)	14.97 (5.55)	16.83 (9.56)	<.001
Hemoglobin level at admission, mean (SD), g/dL	14.03 (1.25)	13.75 (2.92)	10.23 (1.95)	<.001
Platelet count at admission, mean (SD), ×10^3^/µL	280.03 (69.00)	293.00 (151.00)	293.40 (216.00)	.940
Surgery performed, No. (%)				<.001
Laparoscopic cholecystectomy	25 (81)	2 (7)	9 (17)	
Appendectomy	4 (13)	14 (37)	16 (31)	
Dehiscence repair	0 (0)	0 (0)	3 (5)	
Primary perforation closure	2 (6)	10 (26)	8 (16)	
Intestinal resection	0 (0)	11 (30)	13 (26)	
Biliperitoneum	0 (0)	0 (0)	3 (5)	

### Serum sTREM1 and TREM1 expression in cells

**Figure 1.**
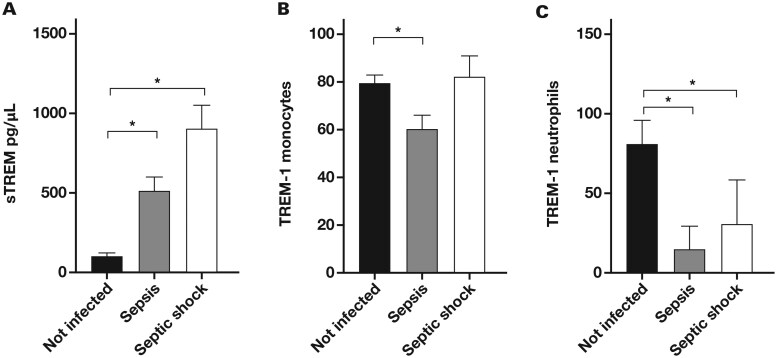
sTREM-1 in sera and TREM-1 in cells. (**A**) Detection of sTREM-1 (mean [SEM], pg/mL) in the sera of patients who underwent abdominal surgery without infection, with sepsis, and with septic shock and the expression of TREM-1 (mean [SEM], %) on the surface of (**B**) monocytes and (**C**) neutrophils. sTREM-1 indicates soluble triggering receptor expressed on myeloid cells 1.

Serum sTREM1 levels progressively increased with disease severity. Noninfected patients exhibited the lowest sTREM1concentrations (mean [SEM], 102 [20] pg/mL), representing a baseline inflammatory state after surgery, which also served as the internal reference value in the absence of manufacturer-established normal ranges. The sepsis group showed a moderate increase (mean [SEM], 517 [80] pg/mL), reflecting an enhanced inflammatory response to infection. In patients with septic shock, sTREM1 levels were markedly elevated (mean [SEM], 908 [146] pg/mL) (Figure 1A), highlighting the systemic inflammation associated with septic shock. Conversely, TREM1 expression on neutrophils declined in patients with sepsis and septic shock, suggesting a potential regulatory adaptation or consumption mechanism associated with infection severity (Figure 1B and 1C).

### sTREM-1 as a biomarker for septic shock

**Figure 2.**
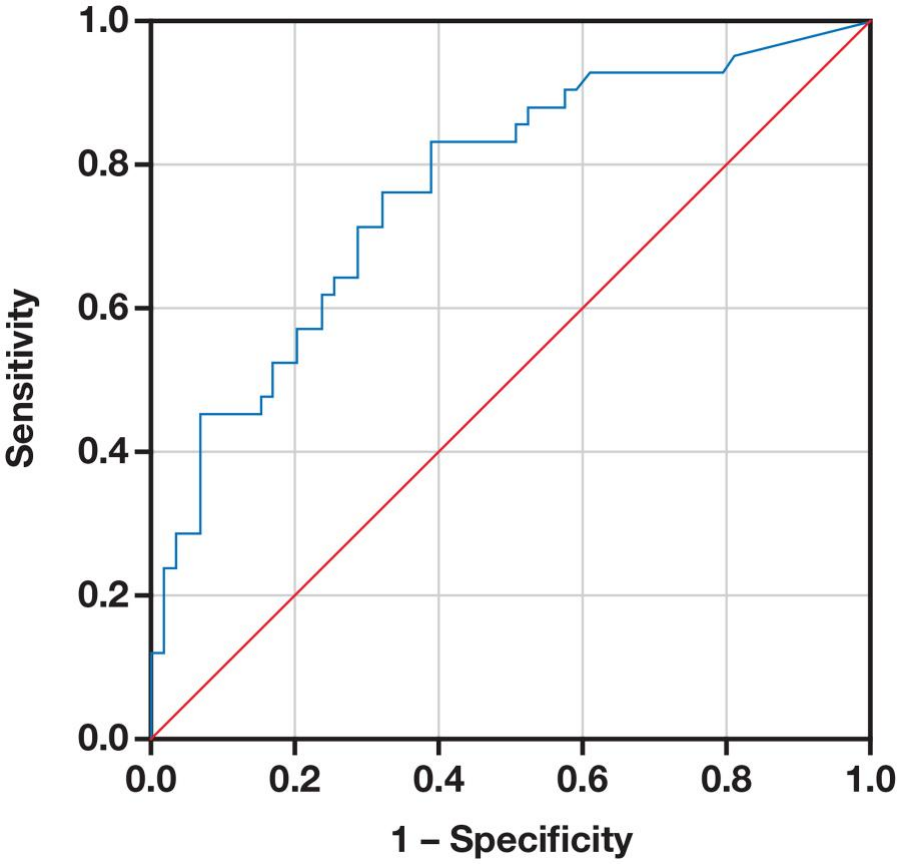
ROC curve of sTREM-1 for the prediction of septic shock. ROC indicates receiver operating characteristic; sTREM-1, soluble triggering receptor expressed on myeloid cells 1.

A ROC curve analysis (Figure 2) was conducted to assess the diagnostic performance of sTREM-1 in predicting septic shock following abdominal surgery. This analysis included only patients with septic shock (*n* = 52) and noninfected surgical patients (*n* = 31), who served as the negative reference group to evaluate the discriminative power of sTREM-1. The area under the curve (AUC) was 0.722 (Figure 2), demonstrating the considerable ability of sTREM-1 to differentiate patients at risk of developing septic shock from patients who were not.

A threshold sTREM-1 concentration of 346 pg/mL was established, optimizing sensitivity (71%) and specificity (69%). Patients with sTREM-1 levels above 346 pg/mL exhibited a statistically significantly increased risk of septic shock. This cutoff value enhances the clinical utility of sTREM-1 by providing a quantitative standard for risk assessment of septic shock in postoperative patients.

### Correlation between SOFA and Brussels scores with sTREM-1

The correlation between serum sTREM-1 levels and SOFA and Brussels scores was evaluated to highlight the prognostic potential of sTREM-1. Among the 52 patients with septic shock, 35 survived and 17 did not. The SOFA and Brussels scores were assessed at admission (day 0), day 2, and day 5, providing a chronological perspective on disease progression.

Nonsurvivors exhibited significantly statistically higher initial SOFA scores (mean [SD], 9.5 [2.8]) compared with survivors (mean [SD], 6.5 [3.2]), a trend that persisted over time, emphasizing the prognostic relevance of SOFA scores in predicting mortality. Similarly, Brussels scores were statistically significantly elevated in nonsurvivors (mean [SD], 8.4 [2.8]) compared with survivors (mean [SD], 5.7 [2.9]) at all-time points, further reinforcing their prognostic value.

A moderate positive correlation was identified between sTREM-1 levels and both SOFA and Brussels scores at all time points, indicating that elevated sTREM-1 levels corresponded with greater organ failure severity and systemic inflammation. This association was statistically significant (*P* values ranging from .001 to .01), underscoring the clinical relevance of sTREM-1 in sepsis assessment (Table 2). The analysis differentiated survivors from nonsurvivors among patients with septic shock, demonstrating that sTREM-1 levels could reflect both current severity and prognosis.

**Table 2.** SOFA and Brussels Scores During 5 Days in Patients With Septic Shock, Survivors and Nonsurvivors, and Correlation of SOFA and Brussels Scores With sTREM-1 in Sera

	Septic shock survivors, mean (SD) (*n* = 35)	Septic shock nonsurvivors, mean (SD) (*n* = 17)	*P* value	sTREM-1, range, pg/mL	Pearson correlation with sTREM-1	***P* value**
SOFA day 0	6.5 (3.2)	9.5 (2.8)	.002	568.03-1089.96	0.41	.001
SOFA day 2	4.0 (3.8)	9.2 (2.4)	.001	331.06-687.06	0.42	.005
SOFA day 5	5.1 (3.0)	10.5 (1.7)	.001	266.86-626.10	0.51	.010
Brussels score day 0	5.7 (2.9)	8.4 (2.8)	.002	568.03-1089.96	0.41	.001
Brussels score day 2	4.3 (3.1)	7.8 (2.1)	.001	331.06-687.06	0.50	.001
Brussels score day 5	4.4 (2.6)	8.8 (1.8)	.001	266.86-626.10	0.61	.002

Abbreviations: SOFA, sequential organ failure assessment; sTREM-1, soluble triggering receptor expressed on myeloid cells 1.

In exploratory analysis of cytokines as an ancillary analysis, we measured selected Th1/Th2 cytokines in a subset of patients to further characterize the immune response. The results, displayed in Supplementary Figure 1, showed differential expression patterns across noninfected patients, patients with sepsis, and patients with septic shock. No correlation was observed, however, between cytokines and sTREM-1 levels (Supplementary Figure S1).

## Discussion

This study examined the role of serum sTREM-1 and TREM-1 expression on cellular surfaces, offering a comprehensive analysis of the inflammatory landscape following abdominal surgery across noninfected, septic, and septic shock patient groups. The findings illustrate the complex interplay between soluble markers and cell-bound indicators of inflammation in postsurgical patients.

The findings of this study underscore the pivotal role of sTREM-1 as a biomarker in sepsis following abdominal surgery. The elevated concentrations of sTREM-1 in patients with sepsis and septic shock compared with individuals without sepsis align with previous research demonstrating its association with inflammation and its potential role as a prognostic indicator of sepsis severity.^17^^,^^18^ The substantial differences in serum sTREM-1 levels across patient groups emphasize its utility in distinguishing varying degrees of septic conditions and establishes sTREM-1 as a potential biomarker for evaluating sepsis progression and severity.

Notably, the ROC analysis in our study yielded an AUC of 0.722, which is lower than values reported in other contexts. This discrepancy may be attributed to differences in patient populations, clinical settings, timing of sample collection, and disease definitions. For example, studies involving broader ICU cohorts or earlier sampling times might report higher AUC values due to more pronounced biomarker kinetics or inclusion of patients with different inflammatory status. Our focus on post–abdominal surgery patients—a population with overlapping postoperative inflammatory responses—might reduce the discriminative power of sTREM-1 in isolation, although it remains clinically valuable when interpreted alongside clinical scores. Despite advances, reliable blood biomarkers of sepsis severity remain limited.

The correlation between elevated sTREM-1 levels and decreased TREM-1 expression on monocytes and neutrophils underscores the intricate dynamics of systemic and cellular immune responses in sepsis and septic shock. These findings contribute to improved diagnostic and prognostic strategies for sepsis and support potential therapeutic interventions targeting the TREM-1 pathway.

The sex distribution in this study, characterized by a higher proportion of male patients in the septic and septic shock groups, suggests that sex may serve as a potential risk factor or indicator of susceptibility to post–abdominal surgery sepsis. This observation aligns with prior studies indicating sex-based differences in immune response and sepsis-related outcomes.^19^

Furthermore, the analysis revealed a notable correlation between sTREM-1 levels and both the SOFA and Brussels scores, reinforcing sTREM-1’s role as a prognostic biomarker. The positive correlation between sTREM-1 concentrations and the severity of organ dysfunction and systemic inflammation provides a quantifiable measure of sepsis severity, which may aid in clinical decision-making. In a supplementary exploratory analysis, we profiled selected cytokines to provide an immunologic context for sTREM-1 elevation. These results revealed distinct cytokine patterns among patient groups; however, no correlation with sTREM-1 levels was identified. The cytokine findings are shown in Supplementary Figure S1 and were not considered central to the primary conclusions of this study.

In conclusion, this study highlights the significance of sTREM-1 as a biomarker for assessing sepsis severity and the risk of septic shock following abdominal surgery. There were correlations between sTREM-1 levels and SOFA and Brussels scores. These findings contribute to a deeper understanding of sepsis pathophysiology and support the development of targeted therapeutic interventions aimed at modulating the immune response to improve patient outcomes.

### Limitations

The sample size of this study was relatively small, however, and limited to a single center, which may affect the generalizability of the findings. In addition, the study population consisted exclusively of adults undergoing abdominal surgery, excluding other patient groups. Future research should involve larger, multicenter cohorts to validate these results and explore the applicability of sTREM-1 across various clinical settings.

Further investigations should focus on elucidating the precise role of sTREM-1 and cytokines in sepsis to enhance diagnostic accuracy and develop more effective therapeutic strategies. In addition, the observed sex differences in sepsis susceptibility warrant further exploration to better understand the underlying mechanisms and their implications for patient management. Cytokine profiling was exploratory and limited by sample size and variability; thus, findings from this component should be interpreted with caution.

## Supplementary Material

lmaf074_Supplementary_Data

## Data Availability

The data underlying this article will be shared on reasonable request to the corresponding author.
