# Supplementary material for: sTREM-1 as a biomarker for sepsis diagnosis and prognosis following abdominal surgery
Source: Lab Med. 2025 Nov 22;57(1):lmaf074. doi: 10.1093/labmed/lmaf074 (PMC12831532; doi:10.1093/labmed/lmaf074)
Supplement: lmaf074_Supplementary_Data [file lmaf074_supplementary_data.pdf]

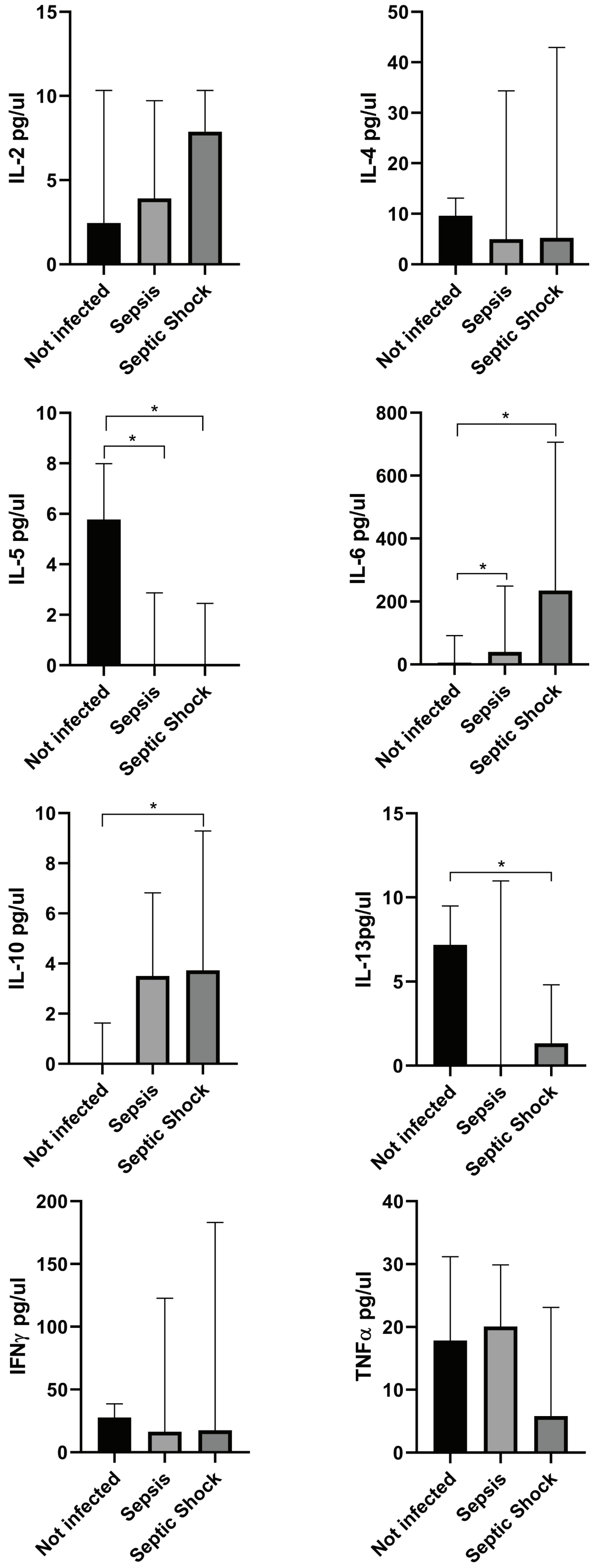

Supplementary Figure S1. Cytokine levels in sera of patients who underwent abdominal surgery: non-infected, sepsis, and septic shock groups. Median values and error bars are shown. *Statistical significance ($P < .05$) indicated where applicable. These results are presented as exploratory data.
